# Cocoa Shell as a Step Forward to Functional Chocolates—Bioactive Components in Chocolates with Different Composition

**DOI:** 10.3390/molecules25225470

**Published:** 2020-11-23

**Authors:** Veronika Barišić, Milica Cvijetić Stokanović, Ivana Flanjak, Kristina Doko, Antun Jozinović, Jurislav Babić, Drago Šubarić, Borislav Miličević, Ines Cindrić, Đurđica Ačkar

**Affiliations:** 1 Faculty of Food Technology Osijek, Josip Juraj Strossmayer University of Osijek, Franje Kuhača 18, 31000 Osijek, Croatia; veronika.barisic@ptfos.hr (V.B.); milica.cvijetic@ptfos.hr (M.C.S.); antun.jozinovic@ptfos.hr (A.J.); jurislav.babic@ptfos.hr (J.B.); drago.subaric@ptfos.hr (D.Š.); bmilicevic@ptfos.hr (B.M.); dackar@ptfos.hr (Đ.A.); 2 Federal Agro Mediterranean Institute, Biskupa Čule 10, 88000 Mostar, Bosnia and Herzegovina; kristina.skender@gmail.com; 3 Department of Agriculture, Polytechnic in Požega, Vukovarska 17, 34000 Požega, Croatia; 4 Department of Food Technology, Karlovac University of Applied Sciences, Trg J.J. Strossmayera 9, 47000 Karlovac, Croatia; icindric@vuka.hr

**Keywords:** dark chocolate, milk chocolate, cocoa shell, CBE, dairy ingredients, polyphenol, flavonoid

## Abstract

Chocolate is considered as both caloric and functional food. Its nutritional properties may be improved by addition of fiber; however, this may reduce polyphenols content. The aim of this research was to determine the influence of cocoa shell addition (as a source of fiber) and its combination with different ingredients (cocoa butter equivalents (CBE), emulsifiers, dairy ingredients) on polyphenols of dark and milk chocolates. Total polyphenol (TPC) and total flavonoid (TFC) contents were determined spectrophotometrically, identification and quantification of individual compounds by high pressure liquid chromatography and antioxidant capacity by ferric reducing antioxidant power (FRAP) assay. Results showed that even though addition of cocoa shell to chocolate results in reduced contents of TPC, TFC, and individual compounds, it is not significant compared to ones reported by other authors for commercial chocolates. Other ingredients influence determined values for all investigated parameters; however, additional research is needed to reveal exact mechanisms and implications.

## 1. Introduction

Chocolate is among the most popular foods worldwide. Regardless of age, ethnicity, social status, health, people have been thriving on it since ancient times. It has evolved from drink to food and transformed from exclusivity to staple food. Along with this transition, the contribution of chocolate to total calorie intake has become an issue, but the beneficial effects of cocoa have emerged as well. To this day, chocolate is considered both as a preferred and unwanted food at the same time. Industry and research communities are seeking solutions to reduce the unwanted characteristics of chocolate (e.g., high energy and sugar content) [1,2] and to improve its polyphenol content [3,4], since this is the major positive characteristic of chocolate [5,6,7].

The aim of this research, however, is to explore the potential of usage of cocoa shell in chocolate production. This is the part of the cocoa bean that is discarded during production, rich in fiber and containing polyphenols of similar profile to cocoa cotyledons and could be used as a source of fiber without major impact on the polyphenolic profile. The main reason why it is not normally used in chocolate production lies in the fact that it causes problems with particle size reduction in traditional processes. This is why legislation in European countries used to limit its content to 5% in cocoa liquor [8]. However, current legislation does not limit its content since technology has evolved, and reuse of food industry by-products in food production is being encouraged. This paper focuses on research into the profile of bioactive components in chocolate with and without cocoa shell, aiming to determine whether replacement of part of the cocoa liquor with the shell would influence these properties. In addition, combinations of cocoa shell with different ingredients, normally used in chocolate production, were investigated as well in order to reveal if there are limitations regarding its use from the aspect of bioactive compounds.

## 2. Results and Discussion

Cocoa shell is being recognized as a valuable raw material in food production as a source of polyphenols [9], fiber [10] or as an antimicrobial agent [11]. Since it is a part of the cocoa bean, one of the major chocolate ingredients, it makes sense to use it in chocolate production. The main ingredient introduced with it into the product is dietary fiber, making up approximately 39–66 g/100 g of the shell [12]. However, the main beneficial chocolate ingredients are polyphenols, with proven health effects, as established by EFSA [6,7] and this should not be disregarded when introducing new ingredients into chocolate.

Total phenolic content (TPC) in chocolates analyzed in this research is presented in Table 1. It ranged from 0.73 mg GAE/g of defatted sample to 1.52 mg GAE/g of defatted sample for milk chocolates, and from 2.41 mg GAE/g of defatted sample to 3.61 mg GAE/g of defatted sample for dark chocolates, as determined by modified Folin-Ciocalteau method. The addition of cocoa shell reduced TPC, with more pronounced effect as cocoa shell content increased from 5% to 15% in dark chocolate; however, TPC in all analyzed samples was larger than reported by Roda and Lambri [13] for dark (2.12 mg GAE/g of defatted sample) and milk (0.64 mg GAE/g of defatted sample) chocolate. Belščak-Cvitanović et al. [14] reported TPC content of approximately 3.5–5.5 mg GAE/g of defatted sample for milk chocolate and approximately 7.5–12 mg GAE/g of defatted sample for dark chocolate. Godočikova et al. [15] reported TPC values of 4.83–23.58 g GAE/kg of defatted sample. In both these researches the standard Folin-Ciocalteu method was applied, corresponding to our results presented as TPC1 in Table 2, which ranged from 1.70 to 3.63 mg GAE/g of defatted sample for milk and 7.54–12.71 mg GAE/g of defatted sample for dark chocolates. It has to be emphasized that these results are overestimated, because when the standard Folin-Ciocalteu method is used, sugars also interact with the Folin-Ciocalteu reagent unlike in the modified method performed in acidic conditions [16].

Total flavonoid content (TFC) in our research ranged from 4.58 to 6.78 µmol CE/g of defatted sample for milk and 9.65–24.62 µmol CE/g of defatted sample for dark chocolates, with decreasing values as cocoa shell content increased both for milk and dark chocolates. These results are in accordance with results for commercial chocolates reported by Todorović et al. [17], with average TFC content of 5.4 ± 0.8 µmol CE/g of defatted sample for milk and 19.1 ± 5.3 µmol CE/g of defatted sample for dark chocolates.

Determined values for TPC and TFC did not only depend on the amount of added cocoa shell, but on other ingredients as well, revealing very complex interactions of polyphenols with other components, for example, carbohydrates, proteins and fats.

When part of the milk powder was replaced with whey (sample MC4 compared to MC3), determined TPC content decreased (from 1.37 to 1.22 mg GAE/g of defatted sample), while TFC content increased (from 4.58 to 5.11 mg GAE/g of defatted sample). Urbanska et al. [18] associated the decrease of polyphenol content to increase of protein content in different milk powders, due to formation of protein micelles entrapping polyphenolic compounds, making them inaccessible. However, the type of protein also makes a difference. Jakobek [19] summarized interactions of polyphenols with proteins, fats and carbohydrates, showing high affinity of (+)-catechin, (−)-epicatechin, (−)-epicatechin gallate and (−)-epigallocatechin gallate for hydrophobic and hydrophilic bonding with ß-lactoglobulin, with higher affinity of heat-treated protein (which is the case in production of whey powder). Hence, lower contents of (+)-catechin, (−)-epicatechin and (−)-epicatechin gallate were determined in milk chocolate produced with whey powder (Table 2). Shpigelman et al. [20] reported that heat (normally applied during production of whey powder) causes unfolding of ß-lactoglobulin, exposing inner parts of protein. These inner parts react with polyphenols via hydrophobic interaction and H-bonding, forming microfibrillar structures. Furthermore, El-Messery et al. [21] reported that the affinity of coffee polyphenols followed the order: skim milk < acid casein < α-casein < β-casein < whey protein isolate < α-lactoglobulin, and Urbanska et al. [18] reported lower contents of polyphenols in milk chocolates produced with milk containing higher levels of proteins, supporting the trend observed in our research.

Fat type also influences determined values for polyphenol content. In this research, 5% of cocoa butter was replaced with palm or coconut oil. In dark chocolates, TPC followed the order: sample without cocoa butter equivalents (CBE) (DC4) > sample with palm oil (DC5) > sample with coconut oil (DC6), while in milk chocolates the order was: sample without CBE (MC3) > sample with coconut oil (MC 6) > sample with palm oil (MC5) (Table 1). A possible explanation could be that part of polyphenols was extracted from samples together with fat during defatting. Jakobek [19] reported that lipids can “capture” polyphenols. The main difference between cocoa butter, palm and coconut oil is in fatty acid composition. While in cocoa butter C18:0 and C18:1 prevail, in palm oil C16:0 is the dominant fatty acid and in coconut oil MCFAs are present, with C12:0 as the most represented [22], which influences polarity of fats. Yara-Varon et al. [23] established vegetable oils, such as corn, olive, palm and refined sunflower oil, as potential green solvents for extraction of polyphenols, as a single solvent, mixed with organic solvents, or combined with different technologies (microwave, ultrasound, supercritical CO2 extraction). Li et al. [24] used different types of oils for extraction of polyphenols from olive leaves. Although castor oil, categorized as polar, had the best performance, good results were achieved with unrefined oils as well. Apparently, minor compounds in oils also affect solubility of polyphenols. Contents of individual polyphenolic compounds followed the order: sample with palm oil (DC5) > sample without CBE (DC4) > sample with coconut oil (DC6) for dark chocolates and sample without CBEs (MC3) > sample with coconut oil (MC6) > sample with palm oil (MC5) for milk chocolates. Apparently, polarity of oil, as well as milk components, influences interactions of fat with polyphenols. According to Li et al. [24], emulsifiers improve extraction of polyphenols with fats. In our research, lecithin was used as a standard emulsifier and part of it was replaced with PGPR (samples MC7 and DC7). This resulted in reduced contents of TPC, CAT, EPI and EPG in milk chocolate, however, the opposite effect was observed for dark chocolate, again implying that milk components interfere with these reactions.

TFC content followed the order: sample with coconut oil > sample with palm oil > sample without CBE. Apparently, solubility of individual compounds in oil and polarity of oils influences the obtained results.

Another component influencing polyphenol behavior in food systems is fiber. In our previous researches we treated cocoa shell with HVED and explored its influence on polyphenols [25] and fiber [26]. HVED reduced contents of all analyzed polyphenolic components and increased content of insoluble fiber, which was reflected in properties of chocolates in this research. Both TPC and TFC were lower in dark chocolates produced with HVED treated cocoa shell, due to initial lower contents (Table 1). This trend, however, is not clearly visible in milk chocolates, where TFC was lower (or equal) in chocolates with untreated cocoa shell. Additionally, when observing particular compounds, correlation between fiber contents and contents of polyphenolic compounds cannot be drawn. In addition to fiber content, porosity and surface properties of fiber, as well as molecular weight of polyphenols and polyphenol-protein complexes influence these interactions [19]. After HVED treatment, both oil- and water-binding capacities of cocoa shell increased [26], indicating that HVED could result in a more porous structure of fiber. The pores could entrap polyphenols not only from cocoa shell, but from cocoa mass as well, making them inaccessible for extraction and determination. Another aspect that has to be taken into consideration is particle size and total surface area for interactions of fiber and polyphenols. Although a specific fraction of cocoa shell was taken into chocolate production, where particle size did not differ between untreated and HVED treated cocoa shell, during milling and conching in ball mill particles were reduced to different extents, depending on chocolate composition. Although parallels cannot be drawn between contents of polyphenolic compounds and specific surface area of chocolate particles (results not shown), it could contribute to complexity of interactions and final availability of polyphenols.

Introducing milk ingredients brings about even more complexity to the system. When 5% of cocoa shell was used (samples MC3 and MC9), addition of shell treated with HVED resulted in lower content of analyzed individual compounds, however, when 2.5% of the shell was used (samples MC2 and MC8), this trend was not observed for all compounds. Specifically, contents of (+)-catechin, (−)-epicatechin and caffeic acid were lower in samples with untreated shell, although the shell itself contained higher proportions, as reported earlier [25]. (−)-Epicatechin is well described in regarding antioxidant properties and reactions involved [27], with more pronounced activity in non-polar media, such as fats (hence, chocolate is a product with high cocoa butter content) [24]. It even interferes with Maillard reactions, quenching sugar fragmentation products [28], could undergo epimerization reactions and finally condensation into tannins also interact with proteins [26]. (+)-Catechin is very similar in properties to (−)-epicatechin and this is the main reason why they show similar behavior in this research as well.

(−)-Epicatechin gallate (EPG) is the compound where it is easiest to see that influence of different ingredients on polyphenol content and availability cannot be easily predicted. Its content in cocoa mass was 0.124 ± 0.007 mg/g, 0.030 ± 0.002 mg/g in untreated cocoa shell and 0.009 mg/g in treated cocoa shell. It is expected that partial replacement of cocoa mass with shell would result in a decrease of EPG in chocolate, but this was not the case. In dark chocolates, EPG content followed the order: DC8 > DC10 > DC9 > DC2 > DC7 > DC3 > DC4 > DC5 > DC1 > DC6 and in milk chocolates the order was: MC3 > MC4 > MC2 > MC7 > MC8 > MC1 > MC9 > MC6 > MC5. EPG is the largest polyphenol of the analyzed molecules with molecular mass of 442.37 g/mol (compared to 290.27 g/mol for (−)-epicatechin and (+)-catechin, 180.16 g/mol for caffeic acid and 164.16 g/mol for *p*-coumaric acid) and the largest number of active sites for interactions with other compounds. It could be trapped within the fiber matrix in cocoa shell and liberated with subsequent milling during chocolate production, or chemically linked to fiber in the shell with disturbance of chemical bonding with increase of hydrophobic conditions in chocolate due to addition of cocoa butter and other fats, or formed through interactions of monomeric compounds, or in combination.

Differences in chemical compositions of chocolates are reflected in their antioxidant properties analyzed by FRAP assay (Table 1). Due to so-called dilution factors, milk chocolates had lower antioxidant capacity. Addition of cocoa shell resulted in a decrease of antioxidant capacity of milk chocolates; however, HVED treatment of cocoa shell prior to addition did not have an influence on the determined values. In dark chocolates, however, the difference between chocolates with HVED treated shell (DC8, 9 and 10) and chocolates without the shell (DC1) and with untreated shell (DC2, 3 and 4) was remarkable, with 30%–50% reduction of determined values. This, however, did not correspond to determined TPC values, but may be linked to the reduction of HMF (5-(hydroxymethyl)furfural) in cocoa shell after HVED treatment determined in our previous research [29]. Although the FRAP assay is considered as a cheap and fast method giving reliable insight into antioxidant capacity, it has been established that different organic and inorganic compounds interfere with the reaction [30,31] and Turkut et al. [32] reported strong positive correlation between HMF content in honey and its antioxidant capacity determined by FRAP. Additionally, the reaction time in assay is too short for complete reaction of all compounds (e.g., caffeic acid) and this could have influenced results as well [33]. Other components (type of CBE, emulsifier, type of dairy protein) did not significantly influence results of antioxidant capacity.

**Figure 1:**
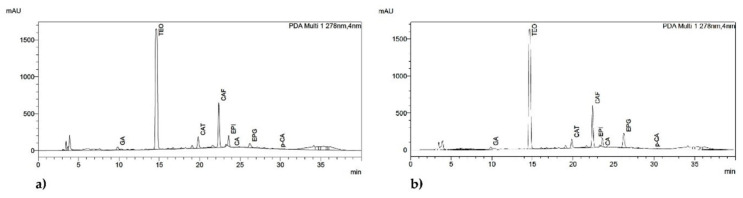
HPLC chromatograms of identified bioactive components in dark chocolate sample (**a**) and dark chocolate with cocoa shell (**b**) recorded at 278 nm. Peaks: GA (gallic acid), TEO (theobromine), CAT ((+)-catechin), CAF (caffeine), EPI ((−)-epicatechin), CA (caffeic acid), EPG ((−)-epicatechin gallate) and *p*-CA (*p*-coumaric acid).

Contents of theobromine in present research were 2.146–3.198 mg/g in milk chocolates and 3.771–4.819 mg/g in dark chocolates. In both cases, the highest content was observed in control samples, while the lowest in samples with the largest contents of HVED treated cocoa shell. Typical chromatograms of dark chocolate sample (DC1) and dark chocolate with cocoa shell (DC10) are presented in Figure 1. Belščak et al. [14] reported significantly higher values of theobromine content both in milk (3.405 mg/g) and in dark chocolates (8.514 mg/g). Batista et al. [34] reported 0.50–12.00 g theobromine/kg chocolate with 70% cocoa, depending on cocoa variety and fermentation conditions, and Bordiga et al. [35] reported values 6.14–8.26 mg/g for dark chocolates. It is evident that contents of theobromine in our research were lower than reported by others. This could be due to differences in raw materials (e.g., cocoa mass) or due to differences in extraction and analyses conditions. The addition of cocoa shell resulted in reductions of theobromine content, but other investigated ingredients did not affect it significantly.

Contents of caffeine in present research were 0.211–0.339 mg/g for milk and 0.562–0.887 mg/g for dark chocolates. Again, it may be observed that the addition of cocoa shell reduced the contents of caffeine, while CBE addition had an influence on its content in milk chocolates. Furthermore, these values are below the ones reported by Belščak-Cvitanović et al. [4] (0.551 mg/g for milk and 0.925 mg/g for dark chocolate), but above the values reported for dark chocolates by Bordiga et al. [35] (0.164–0.347 mg/g).

Methylxanthines in cocoa and cocoa products do not only pose health effects through action on adenosine receptors in the central nervous system and boosting concentration, mood and arousal levels, but have an impact on sensory properties of chocolate as well [36], and their contents are important both from the aspect of quality and for functional properties of chocolate.

## 3. Materials and Methods

### 3.1. Chemicals

Hydrochloric acid, n-hexane (HPLC grade) and Folin-Ciocalteu reagent were purchased from Carlo Erba Reagents (Val de Reuil, France), and sodium hydroxide and sodium nitrite from Gram-mol (Zagreb, Croatia), ferric chloride hexahydrate and sodium acetate trihydrate from T.T.T. (Sveta Nedelja, Croatia), and aluminum chloride hexahydrate from Kemika (Zagreb, Croatia). Sodium carbonate was supplied by Panreac Química (Castellar del Vallès, Spain), ferrous sulfate heptahydrate by Acros Organics (Madrid, Spain) and 2,4,6-Tris(2-pyridyl)-s-triazine (TPTZ) by Alfa Aesar (Karlsruhe, Germany). HPLC grade standards of theobromine, caffeine and (+)-catechin, (−)-epicatechin gallate, caffeic acid and *p*-coumaric acid, gallic acid and (−)-epicatechin were purchased from Sigma-Aldrich (St. Louis, MO, USA). Methanol (J.T. Baker, Deventer, The Netherlands) and formic acid (Scharlau Chemie, Spain) used for mobile phase preparation were HPLC grade.

### 3.2. Chocolate Samples

Cocoa mass and cocoa butter were purchased from DGF (Paris, France), milk powder from Dukat d.d. (Zagreb, Croatia), whey powder from Vindija d.d. (Varaždin, Croatia), powdered soy lecithin from A.C.E.F. (Fiorenzuola d’Arda, Italy), polyglycerol polyricinoleate (PGPR) from (Azelis, Croatia), palm oil from Rapunzel (Legau, Germany) and coconut oil from Zvijezda d.d. (Zagreb, Croatia).

Dark and milk chocolates were produced in a laboratory ball mill with different amounts of untreated cocoa shell (roasted at 135 °C, 55 min) and cocoa shell treated with high voltage electrical discharge (HVED) (Table 3). Treated cocoa shell was subjected to 15 kV/cm of electric field density at concentration of 3%, frequency of 40 Hz and time of 15 min. Treated shell was frozen at −80 °C and freeze-dried (Alpha LCS Plus, Christ, Osterode am Harz, Germany). Untreated and treated cocoa shell were milled (M20, IKA, Staufen, Germany) and sieved, after which the portion under 71 µm was used in production. For dark chocolates 2.5 kg of stainless steel balls (9.525 mm diameter) were used in production and 3 kg for milk. Mixing was performed at 60 rpm and temperature was controlled with water bath (55 °C). Dark and milk chocolates without added cocoa shell were mixed for 3 h and the ones with added shell for 3.5 h (fats and cocoa shell were added half an hour before other ingredients). Emulsifiers were added one hour before end of mixing, and aroma (vanillin) half an hour before the end of mixing in ball mill. Tempering was carried out by hand and the temper index was measured with a Sollich Tempermeter E3 (Bad Salzuflen, Germany) (desired temper index 4–7). After tempering, chocolates were molded and cooled for 30 min at 8 °C.

### 3.3. Extraction of Bioactive Components

Extraction of bioactive components from chocolate samples was performed according to Adamson et al. [37] and Barišić et al. [25]. Prior to the extraction, chocolates were grated and 3 g of grated sample was treated three times with 10 mL of n-hexane to eliminate lipids. The remaining defatted solids were air-dried for 24 h. Afterwards, the extraction procedure of bioactive components was as follows: two grams (±0.1 g) of defatted chocolate sample was mixed with 5 mL of 70% aqueous methanol, sonicated for 30 min, centrifuged for 10 min at 3000 rpm and the supernatant was decanted into a 10 mL volumetric flask. The extraction procedure in an ultrasonic bath and centrifugation was repeated once more with the same amount of 70% aqueous methanol. After combining the extracts, the volumetric flask was filled up to the mark with 70% aqueous methanol. The obtained extracts were stored in a freezer and filtered through 0.45 μm nylon membrane filter before analyses.

### 3.4. HPLC Analysis

Selected methylxanthines and phenolics were identified and quantified as described in our previous paper [25]. The analysis was performed on a Shimadzu HPLC instrument equipped with a LC-20AD quaternary pump (Shimadzu, Kyoto, Japan), a CTO-20AC column oven (Shimadzu, Kyoto, Japan), a SPD-M20A array detector (PDA, Shimadzu, Kyoto, Japan) and a SIL-10AF autosampler (Shimadzu, Kyoto, Japan). Separation of components was performed using an Inertsil ODS-3V column (GL Sciences, Tokyo, Japan) (250 × 4.6 cm, 5 μm particle size). The gradient elution (flow rate 0.8 mL/min) was performed using HPLC grade methanol (solvent A) and 1% formic acid (solvent B). Starting percentage of solvent A in the mobile phase was 10%, followed by linear increase to 32% A at 15 min, 40% A at 20 min up to 25 min and 60% A at 30 min. The injection volume of the sample was 20 μL, and the column and detector temperatures were 30 °C. Spectrum monitoring was performed in the wavelength range from 200 to 400 nm, while detection of separated components was at 278 nm. Identification of bioactive components was achieved based on the comparison of the retention times and spectrum data with those of the standards while quantification was obtained with the external calibration method. All analyses were repeated three times, and the results expressed as mg of specific bioactive component per g of defatted chocolate (mg/g).

### 3.5. Total Phenolic Content

Total phenolic content of chocolate extract was determined by two methods, the original Folin-Ciocalteu method (TPC1) [38] and by modified Folin-Ciocalteu method (TPC2) [16]. All analyses were performed in triplicate.

Total phenolic content (TPC1) was determined as follows: an aliquot (0.1 mL) of chocolate extract was mixed with 6 mL of water and 0.5 mL of Folin-Ciocalteu reagent in the volumetric flask. After 6 min, 1.5 mL of 20% Na_2_CO_3_ was added and the flask was filled up with distilled water to final volume (10 mL). The prepared mixture was left for 2 h at room temperature in a dark place and the absorbance of final solution was measured at 760 nm against the blank. The quantification using gallic acid as standard (0.14–1.0 mg/mL) and the results were expressed as mg gallic acid equivalents per g of defatted chocolate (mg GAE/g).

The modified Folin-Ciocalteu method [16] performed under acidic conditions (without addition of Na_2_CO_3_) was used to eliminate sugar interference with the Folin-Ciocalteu reagent. Briefly, 0.1 mL of chocolate extract was mixed with 1 mL of 10% Folin-Ciocalteu reagent. After vortexing for 2 min, the mixture was incubated for 20 min at room temperature in the dark. The absorbance was determined at 750 nm against the blank. The quantification was carried out using gallic acid as standard (0.14–0.70 mg/mL) and the results were expressed as mg gallic acid equivalents per g of defatted chocolate (mg GAE/g).

### 3.6. Total Flavonoid Content

The determination of total flavonoid content (TFC) was performed according to the Yang et al. [39] with minor modifications. An aliquot of methanolic chocolate extract (0.125 mL) was mixed with 1.5 mL of water and 0.15 mL of 5% NaNO_2_. After 5 min of incubation at room temperature, 0.75 mL 2% AlCl_3_·6H_2_O was added. After 5 min of incubation, 1 mL of 1 M NaOH was added and the mixture filled with distilled water up to final volume (5 mL). The absorbance of reaction mixture was measured at 510 nm against the blank. Total flavonoid content, expressed as µmol catechin equivalents per g of defatted chocolate (µmol CE/g), was calculated from a calibration curve (0.1–3.5 mM) of catechin standard solutions. All measurements were performed in triplicate.

### 3.7. FRAP (Ferric Reducing Antioxidant Power) Assay

For the determination of total antioxidant capacity, the assay described by Benzie and Strain [40] was used. The working FRAP reagent was prepared daily by mixture of 10 mM TPTZ (2,4,6-Tris(2-pyridyl)-s-triazine) solution dissolved in 40 mM HCl, 20 mM FeCl_3_ and 0.3 M acetate buffer solution (pH 3.6) in 1:1:10 ratio. An aliquot of 0.1 mL diluted chocolate extract was mixed with 1.9 mL of FRAP reagent, vortexed and incubated at 37 °C for 4 min. After incubation, the absorbance was measured at 593 nm against the blank. All measurements were performed in triplicate. Aqueous standard solutions of FeSO_4_·7H_2_O (0.1–1 mM) were used for calibration curve and the results were expressed as the FRAP value (μmol Fe(II)/g of defatted chocolate).

## 4. Conclusions

The research presented in this paper shows that cocoa shell may be used in chocolate production as a source of dietary fiber, without major impact on polyphenol and methylxanthine contents even though it does negligibly reduce the contents of these bioactive compounds. It may be combined with commonly used CBEs and emulsifiers, as well as milk components. It is yet to be revealed exactly how CBEs and different proteins interact with polyphenolic compounds and how these interactions influence bioavailability of the bioactive compounds in chocolates.

## Figures and Tables

**Table 1:** Total phenolic (TPC1 and TPC2) and flavonoid (TFC) content and antioxidant capacity (FRAP) of analyzed chocolate samples (average ± standard deviation).

Sample	TPC1	TPC2	TFC	FRAP Value
	mg GAE/g	mg GAE/g	µmol CE/g	µmol Fe(II)/g
MC1	3.63 ± 0.22	1.52 ± 0.02	6.78 ± 0.04	33.96 ± 0.25
MC2	2.61 ± 0.32	1.07 ± 0.13	5.72 ± 0.10	31.46 ± 0.31
MC3	3.18 ± 0.07	1.37 ± 0.09	4.58 ± 0.21	27.10 ± 0.08
MC4	2.92 ± 0.07	1.22 ± 0.05	5.11 ± 0.03	28.08 ± 1.10
MC5	2.35 ± 0.17	1.05 ± 0.04	4.94 ± 0.06	25.17 ± 0.35
MC6	2.78 ± 0.14	1.12 ± 0.06	5.05 ± 0.06	24.67 ± 0.71
MC7	2.71 ± 0.08	1.12 ± 0.06	5.21 ± 0.05	26.85 ± 0.73
MC8	3.09 ± 0.08	1.28 ± 0.04	5.72 ± 0.15	30.86 ± 0.25
MC9	1.70 ± 0.23	0.73 ± 0.07	4.62 ± 0.06	27.04 ± 0.87
DC1	12.71 ± 0.11	3.61 ± 0.04	24.62 ± 0.48	108.53 ± 2.23
DC2	10.86 ± 0.09	3.33 ± 0.04	18.25 ± 0.27	110.23 ± 0.82
DC3	8.95 ± 0.44	2.85 ± 0.07	15.22 ± 0.30	90.80 ± 0.50
DC4	8.87 ± 0.61	2.70 ± 0.16	11.74 ± 0.48	75.04 ± 0.98
DC5	8.84 ± 0.28	2.63 ± 0.02	12.18 ± 0.17	61.26 ± 1.03
DC6	7.82 ± 0.14	2.41 ± 0.04	12.60 ± 0.34	74.92 ± 0.90
DC7	8.57 ± 0.19	2.72 ± 0.11	10.99 ± 0.13	77.37 ± 0.80
DC8	10.68 ± 0.50	3.20 ± 0.04	13.80 ± 0.21	53.10 ± 0.24
DC9	7.94 ± 0.11	2.71 ± 0.05	13.24 ± 0.36	53.47 ± 0.21
DC10	7.54 ± 0.40	2.43 ± 0.14	9.65 ± 0.63	50.65 ± 0.72

**Table 2:** The content of methylxanthines and phenolic components in analyzed chocolate samples (average ± standard deviation).

Sample	Bioactive Component (mg/g)							
	TEO	CAF	CAT	EPI	EPG	GA	CA	*p*-CA
MC1	3.198 ± 0.139	0.339 ± 0.028	0.237 ± 0.019	0.242 ± 0.019	0.215 ± 0.018	0.014 ± 0.001	0.004 ± 0.001	0.014 ± 0.000
MC2	2.799 ± 0.171	0.287 ± 0.024	0.165 ± 0.012	0.171 ± 0.012	0.338 ± 0.040	0.011 ± 0.001	0.002 ± 0.000	0.014 ± 0.000
MC3	3.056 ± 0.009	0.338 ± 0.004	0.183 ± 0.003	0.184 ± 0.002	0.410 ± 0.040	0.011 ± 0.000	0.004 ± 0.000	0.013 ± 0.001
MC4	2.932 ± 0.030	0.304 ± 0.003	0.167 ± 0.002	0.173 ± 0.002	0.364 ± 0.029	0.014 ± 0.001	0.004 ± 0.000	0.009 ± 0.005
MC5	2.843 ± 0.041	0.270 ± 0.004	0.149 ± 0.004	0.154 ± 0.004	0.182 ± 0.003	0.010 ± 0.000	0.002 ± 0.000	0.013 ± 0.000
MC6	2.868 ± 0.057	0.288 ± 0.004	0.162 ± 0.004	0.166 ± 0.005	0.185 ± 0.024	0.011 ± 0.000	0.003 ± 0.001	0.014 ± 0.002
MC7	2.992 ± 0.071	0.311 ± 0.010	0.167 ± 0.007	0.176 ± 0.005	0.304 ± 0.063	0.010 ± 0.000	0.004 ± 0.000	0.012 ± 0.000
MC8	3.029 ± 0.068	0.327 ± 0.015	0.202 ± 0.009	0.204 ± 0.009	0.244 ± 0.021	0.010 ± 0.001	0.004 ± 0.000	0.014 ± 0.001
MC9	2.146 ± 0.100	0.211 ± 0.011	0.113 ± 0.006	0.120 ± 0.008	0.201 ± 0.016	0.005 ± 0.001	0.000 ± 0.000	0.013 ± 0.001
DC1	4.819 ± 0.082	0.887 ± 0.079	0.667 ± 0.065	0.701 ± 0.073	0.272 ± 0.033	0.042 ± 0.005	0.032 ± 0.005	0.024 ± 0.004
DC2	4.621 ± 0.042	0.856 ± 0.012	0.596 ± 0.008	0.613 ± 0.015	0.346 ± 0.013	0.041 ± 0.001	0.028 ± 0.002	0.023 ± 0.001
DC3	4.326 ± 0.016	0.738 ± 0.045	0.464 ± 0.030	0.471 ± 0.032	0.295 ± 0.029	0.039 ± 0.002	0.021 ± 0.002	0.017 ± 0.000
DC4	4.171 ± 0.066	0.653 ± 0.026	0.379 ± 0.027	0.384 ± 0.019	0.289 ± 0.019	0.035 ± 0.001	0.019 ± 0.001	0.018 ± 0.001
DC5	4.221 ± 0.023	0.669 ± 0.008	0.410 ± 0.015	0.417 ± 0.005	0.273 ± 0.016	0.039 ± 0.004	0.024 ± 0.001	0.020 ± 0.000
DC6	4.043 ± 0.013	0.614 ± 0.015	0.355 ± 0.008	0.362 ± 0.007	0.237 ± 0.006	0.032 ± 0.001	0.017 ± 0.000	0.017 ± 0.000
DC7	4.103 ± 0.071	0.690 ± 0.007	0.394 ± 0.006	0.404 ± 0.012	0.335 ± 0.016	0.039 ± 0.001	0.020 ± 0.002	0.018 ± 0.001
DC8	4.568 ± 0.061	0.812 ± 0.031	0.553 ± 0.018	0.561 ± 0.029	0.431 ± 0.036	0.033 ± 0.001	0.023 ± 0.003	0.021 ± 0.005
DC9	4.665 ± 0.528	0.683 ± 0.032	0.393 ± 0.027	0.473 ± 0.050	0.382 ± 0.044	0.030 ± 0.007	0.027 ± 0.010	0.022 ± 0.010
DC10	3.771 ± 0.106	0.562 ± 0.036	0.319 ± 0.021	0.280 ± 0.020	0.403 ± 0.037	0.019 ± 0.002	0.000 ± 0.000	0.013 ± 0.000

TEO, theobromine; CAF, caffeine; CAT-(+), catechin; EPI-(−), epicatechin; EPG-(−), epicatechin gallate; GA, gallic acid; CA, caffeic acid; *p*-CA, *p*-coumaric acid.

**Table 3:** Composition of chocolate samples.

Sample	Cocoa Mass (%)	Cocoa Butter (%)	Milk Powder (%)	Untreated Cocoa Shell (%)	Treated Cocoa Shell (%)	Lecithin (%)	Footnote
MC1	14.74	24.83	15.0	-	-	0.4	
MC2	12.24	24.83	15.0	2.5	-	0.4	
MC3	9.74	24.83	15.0	5.0	-	0.4	
MC4	9.74	24.83	10.0	5.0	-	0.4	+5% whey
MC5	9.74	19.83	15.0	5.0	-	0.4	+5% palm oil
MC6	9.74	19.83	15.0	5.0	-	0.4	+5% coconut oil
MC7	9.74	24.83	15.0	5.0	-	0.2	+0.2% PGPR
MC8	12.24	24.83	15.0	-	2.5	0.4	
MC9	9.74	24.83	15.0	-	5.0	0.4	
DC1	36.00	21.57	-	-	-	0.4	
DC2	31.00	21.57	-	5.0	-	0.4	
DC3	26.00	21.57	-	10.0	-	0.4	
DC4	21.00	21.57	-	15.0	-	0.4	
DC5	21.00	16.57	-	15.0	-	0.4	+5% palm oil
DC6	21.00	16.57	-	15.0	-	0.4	+5% coconut oil
DC7	21.00	21.57	-	15.0	-	0.2	+0.2% PGPR
DC8	31.00	21.57	-	-	5.0	0.4	
DC9	26.00	21.57	-	-	10.0	0.4	
DC10	21.00	21.57	-	-	15.0	0.4	

All milk chocolates had 45% sugar and dark chocolates had 42% sugar; all chocolates had 0.03% vanillin. PGPR-polyglycerol polyricinoleate.
